# Analytical Performance of a Multiplexed Microarray Assay for Rapid Identification and Quantification of a Multivalent mRNA Vaccine

**DOI:** 10.3390/vaccines12101144

**Published:** 2024-10-05

**Authors:** Megan N. Gerold, Evan Toth, Rebecca H. Blair, Rachel Y. Gao, Durgesh V. Nadkarni, Sutapa Barua, Joshua Woods, Kathy L. Rowlen, Erica D. Dawson

**Affiliations:** 1 InDevR Inc., 6035 Longbow Dr, Suite 102, Boulder, CO 80301, USA; 2 BioTherapeutics Pharmaceutical Sciences, Bioprocess Research & Development, Pfizer, Inc., 875 Chesterfield Parkway West, Chesterfield, MO 63017, USA; 3 BioTherapeutics Pharmaceutical Sciences, Pharmaceutical Research & Development, Pfizer Inc., One Burtt Road, Andover, MA 01810, USA; 4 BioTherapeutics Pharmaceutical Sciences, Analytical Research & Development, Pfizer, Inc., 875 Chesterfield Parkway West, Chesterfield, MO 63017, USA

**Keywords:** multivalent mRNA, quantification, identity, nucleic acid, microarray

## Abstract

mRNA vaccines were highly effective in response to the COVID-19 pandemic, making them an attractive platform to address cancers and other infectious diseases. Many new mRNA vaccines in development are multivalent, which represents a difficulty for the standard assays commonly used to characterize the critical quality attributes of monovalent formulations. Here, we present a multiplexed analytical tool with nucleic acid microarray technology using the VaxArray platform that measures the identity and quantity of mono- and multivalent mixtures of naked mRNA and mRNA encapsulated in lipid nanoparticle formulations in under 2 h without any additional preparation steps, such as extraction or RT-PCR. Using a quadrivalent mixture of encapsulated mRNA constructs that encode for four unique proteins in a vaccine formulation, the VaxArray mRNA assay was demonstrated to be highly specific for each mRNA with sensitivity < 1 µg/mL. The quantification of individual mRNAs within the lipid nanoparticle mixture resulted in a precision of ≤10% RSD and an accuracy of 100 ± 9%.

## 1. Introduction

mRNA vaccines were critical in reducing morbidity and mortality throughout the COVID-19 global pandemic [1,2,3,4,5]. The first two COVID-19 mRNA vaccines to gain Emergency Use Authorization, COMIRNATY (Pfizer-BioNTech) [6,7] and SpikeVax (Moderna), resulted in high safety and efficacy against SARS-CoV-2 and were developed quite rapidly compared to any other vaccines in recent history [8,9,10]. Since the 2020 pandemic, mRNA vaccines have been the subject of an increasing number of clinical trials [9,11,12], with most major vaccine manufacturers having active development pipelines for future mRNA vaccines. In addition, mRNA vaccine formulations targeting multiple antigens are increasing in importance, including a recent experimental influenza mRNA vaccine formulation with mRNAs encoding for 20 unique hemagglutinin antigens (HAs) that elicited an immune response in mice and ferrets [13].

Although specific manufacturing processes for mRNA vaccines vary by manufacturer, the same manufacturing platform can be used for a variety of mRNA vaccine targets; therefore, once the target protein sequence is identified, mRNA vaccines can be manufactured quickly at a clinical and commercial scale. Further, as mutations arise in the pathogen of interest due to antigenic drift and selective immune pressure, such as for RNA viruses like influenza and coronavirus, modifications can easily be made in the coding sequence of the mRNA construct(s) to create updated vaccines. Since the approval of the initial COMIRNATY and SpikeVax monovalent vaccines for SARS-CoV-2, there have been on-market multivalent mRNA vaccines as well as ongoing clinical trials for multivalent vaccines targeting multiple subtypes or variants of a single pathogen [14,15,16], including bivalent COVID-19 mRNA vaccines [14,15], multivalent influenza vaccines targeting 4 hemagglutinin (HA) [14,16] proteins and neuraminidase (NA) proteins [16,17], and combination seasonal influenza/COVID-19 vaccine targets [14,16]. 

mRNA comprises (5′ to 3′) a 5′ cap, a 5′ untranslated region (UTR), the coding sequence for the antigen of interest, a 3′ UTR, and a 3′ polyA tail [18,19]. The 5′ cap and 3′ polyA tail allow for efficient translation, and the UTRs are designed to optimize protein expression [19]. A single mRNA molecule can be designed to code for multiple peptides [9]; alternatively, multiple mRNAs that each encode for a different protein can be mixed to create a multivalent vaccine formulation. The mRNA(s) is encapsulated in a lipid nanoparticle (LNP) to stabilize the mRNA, enable cellular uptake of the mRNA(s) for protein expression [19], and improve the in vivo stability of mRNA. 

Analytics for measuring mRNA critical quality attributes (CQAs) such as identity, concentration, and integrity throughout the vaccine manufacturing process are emerging and evolving, but many existing tools are inherently singleplex and therefore not compatible with characterizing multivalent mRNA and may not be optimal for characterizing mRNA encapsulated in LNPs [20,21,22,23]. The absorbance-based quantification of nucleic acids can be performed quickly and easily using UV spectroscopy for naked mRNA or via the fluorescence-based RiboGreen assay for mRNA encapsulated in LNPs [23]. However, absorbance-based methods can only measure total mRNA content and cannot provide information about integrity or distinguish the concentration of each unique mRNA in a multivalent mixture. The two most common analytical techniques for mRNA identity are sequencing (NGS, Sanger) and RT-PCR. While both sequencing and RT-PCR can confirm the identity of mRNA constructs in multivalent mixtures, an often lengthy extraction is required prior to the analysis of encapsulated mRNAs. Multivalent quantification can also be performed using RT-qPCR and RT-dPCR, but again, an extraction step is required for mRNA encapsulated in LNPs. 

A multiplexed nucleic acid microarray technology based on the VaxArray platform (InDevR, Inc., Boulder, CO) was developed to simplify mRNA vaccine quantification and identification during the manufacturing process [24]. A VaxArray microarray for mRNA detection comprises synthetic DNA oligonucleotides designed to specifically capture the mRNA(s) of interest. The captured mRNAs are subsequently labeled via a variety of detection label approaches, and the fluorescence signal of the bound and labeled mRNA is proportional to the concentration of mRNA in the sample. The platform provides rapid (<2 h) identification and quantification of the mRNA material and can quantify multivalent material at multiple stages of the bioprocess (both naked RNA and encapsulated RNA). Once a new mRNA sequence is designed, it only takes 4–6 weeks to update and validate the capture sequences on the microarray.

In our previous work [24], we demonstrated proof of concept for a VaxArray mRNA assay using model influenza HA and NA coding region constructs from the literature using commercially manufactured mRNA [25]. Here, we expand on this initial work by assessing the analytical performance metrics of a VaxArray mRNA assay on an actual quadrivalent mRNA vaccine formulation, namely, four unique, full-length Pfizer-designed mRNA constructs targeting four different proteins. The work herein focuses on assessment of mRNA encapsulated in LNPs, which were analyzed in monovalent samples and as a multivalent formulation, assessing specificity, sensitivity, precision, and accuracy to highlight the utility of the platform for mRNA identification and quantification on a vaccine-relevant mRNA formulation. 

## 2. Materials and Methods

### 2.1. mRNA Constructs

Four monovalent mRNAs, designated as mRNA A, B, C, and D, encoding for four different proteins, were provided by Pfizer as both naked mRNAs and mRNAs encapsulated in LNPs. Each construct included UTRs on both the 3′ and 5′ ends, a 5′ cap, and a 3′ polyA tail. The concentrations of each sample were provided by Pfizer (Chesterfield, MO, USA), and the concentrations of the monovalent naked mRNAs were confirmed in-house by UV spectroscopy.

### 2.2. Oligonucleotide Sequence Design

Capture oligonucleotide (oligo) sequences (~20 mers) were designed to be complimentary to unique regions of each mRNA construct. These regions were designed to provide specificity to the four constructs and were identified by aligning the four mRNA construct sequences in BioEdit (v7.2.5) and then further evaluated with OligoAnalyzer^TM^ (IDT; Coralville, IA, USA). Capture oligos were designed to have melting temperatures > 40 °C to enable room temperature hybridization and self-dimer potential ΔG ≥ 5.5 kcal/mol. Oligos were also evaluated in silico against off-target mRNA constructs to anticipate non-specific interactions. A total of 113 capture oligos were designed and purchased from IDT (Coralville, IA, USA) at high purity: 34 targeting mRNA A, 33 targeting mRNA B, 22 targeting mRNA C, and 23 targeting mRNA D. In addition, an HPLC-purified detection label targeting the polyA tail was synthesized by IDT and included a 5′ Cy3 fluorophore modification to enable fluorescence detection with the VaxArray Imaging System (InDevR, Inc., Boulder, CO, USA).

### 2.3. Microarray Printing and Initial Down-Selection

Capture oligos were printed onto epoxide-functionalized glass slides at InDevR using a piezoelectric microarray printing system. Each slide contained 16 replicate microarrays. All capture oligos were initially evaluated for reactivity and specificity with each naked mRNA construct.. Eleven capture oligos (2–3 per mRNA construct and the polyA control) were then down-selected, based on high specificity for the mRNA construct of interest. For the testing reported here, each capture oligo was printed in triplicate at three different locations (a total of nine spots) throughout the microarray. All coding region capture oligos were used to assess assay performance, whereas the polyA capture oligo was used as a control to detect the presence of any mRNA construct containing a 3′ polyA tail. Of the coding region capture oligos assessed, the B(ii), D(i), C(ii), and A(i) capture oligos had the best overall performance for the naked and encapsulated mRNA sample types, and therefore performance metrics for these specific oligos are presented here. 

### 2.4. Assay Procedure for mRNA Encapsulated in LNPs 

The VaxArray slides, 2x mRNA Oligo Binding Buffer (VXI-6316, InDevR Inc.), and 10% Zwittergent 3–14 (693017-25GM, EMD Millipore Corp., Billerica, MA, USA) were allowed to come to room temperature for at least 30 min prior to testing. Each incubation step was performed at room temperature with the slide(s) inside of a humidity chamber (VX-6204, InDevR Inc., Boulder, CO, USA) while shaking on an orbital shaker at 80 RPM. Slides were pre-washed by adding 50 µL of mRNA Wash Buffer 1 (VXI-6317, InDevR Inc.) to each well and incubated for 1 min. Calibrants and/or samples were diluted in 1x mRNA Oligo Binding Buffer (VX-6316, InDevR Inc.) to a final 1x concentration with 2% Zwittergent 3–14. First, 50 µL of the sample was added to each well of the VaxArray slide and incubated for 1 hr. The slides were then washed with 50 µL of mRNA Wash Buffer 1 in the same manner as the pre-wash. The detection label was diluted to a final concentration of 1x mRNA Oligo Binding Buffer, and 50 µL was added to the slide and incubated for 30 min. The slides were then placed inside a wash bin (VX-6206, InDevR Inc.) containing mRNA Wash Buffer 1 and inverted 15 times. The liquid was removed from the wash bin and replaced with mRNA Wash Buffer 2 (VXI-6318, InDevR Inc.). Without inverting, Wash Buffer 2 was quickly removed and replaced with mRNA Wash Buffer 3 (VXI-6319, InDevR Inc.) and inverted 15 times. The slides were removed from the wash bin, and excess liquid within the wells was removed with a pipette. The slides were then spun dry with the ArrayDry instrument (VX-6218, InDevR Inc.) for at least 10 s, or until wells were visibly dry. Slides were imaged on the VaxArray Imaging System (VX-6000, InDevR Inc.). Samples were quantified against a matched calibration curve, i.e., a naked calibration curve with naked samples or an encapsulated mRNA calibration curve with encapsulated mRNA samples. Data analysis was performed using the VaxArray Imaging System Software, version 3. 

### 2.5. Encapsulated mRNA Reactivity and Specificity

The specificity of each capture oligo was evaluated by testing a replicate set (n = 4) of each monovalent encapsulated mRNA construct at a concentration of 5 µg/mL. A capture oligo was considered specific if the signal-to-background (S/B) ratio with the on-target mRNA construct was ≥3, and the S/B ratio of any off-target mRNA construct was <3. 

### 2.6. Monovalent versus Multivalent Comparison of mRNAs Encapsulated in LNPs

A 7-point dilution series of each encapsulated mRNA construct plus a buffer-only blank was prepared from 0.25 to 5 µg/mL for both monovalent and quadrivalent LNP formulations. For each capture oligo, the background-subtracted signal (S-B, in relative fluorescence units or RFUs) was plotted as a function of mRNA concentration in µg/mL. The fit for every standard curve was chosen based on the best fit based on the shape of the curve; therefore, each regression was specific to the concentration range of the standard curve for each construct.

### 2.7. Limits of Quantification for mRNAs Encapsulated in LNPs

To determine the lower limit of quantification (LLOQ) of the assay for each mRNA construct, four replicates of a quadrivalent mixture were run at several dilutions near the expected LLOQ. These replicates were quantified against a 7-point calibration curve and a blank prepared from 0.025 to 1.5 µg/mL of each encapsulated mRNA construct and fit to a quadratic regression with an R^2^ ≥ 0.99. The LLOQ was defined as the lowest concentration tested for which the sample replicates had a precision of ≤20% relative standard deviation (%RSD) and an accuracy of 100% ± 20% expected. Precision (%RSD) was calculated by dividing the standard deviation of the back-calculated concentrations of the four replicates by the average back-calculated concentration of the same replicates and expressed as a percentage. Accuracy (% expected) was calculated by dividing the average experimental back-calculated concentration of the four replicates by the theoretical concentration of the replicates (expected concentration based on dilution), expressed as a percentage.

The upper limit of quantification (ULOQ) of each mRNA construct was determined in a similar manner to the LLOQ, with sample replicates run at concentrations near the estimated upper limit. The calibration curves were prepared from 1.25 to 25 µg/mL of each mRNA construct, analyzed, and fit to a 4-parameter logistic (4PL) regression with an R^2^ ≥ 0.99. The ULOQ for each construct was defined as the highest concentration for which the precision of the sample replicates was ≤20% RSD, and the accuracy was 100% ± 20% expected. 

The quantification range of each capture oligo was defined as the concentration at the ULOQ divided by the concentration at the LLOQ.

### 2.8. Precision and Accuracy Testing for mRNAs Encapsulated in LNPs 

Precision and accuracy testing was performed on three microarray slides. A quadrivalent, 8-point calibration curve comprising 7 standards was prepared from 0.25 to 5 µg/mL of encapsulated mRNA plus a buffer-only blank. This curve was used to quantify 8 replicates per slide of the quadrivalent mixture at 2 µg/mL (24 replicates total). All calibration curves were fit to a quadratic regression, except for D(i), which was fit to a 4PL regression. All calibration curve regressions had an R^2^ ≥ 0.99.

### 2.9. Assay Procedure for Unencapsulated mRNA

Naked mRNA testing was conducted using the same assay procedure used for the encapsulated mRNA, excluding the Zwittergent 3–14 added to the binding buffer. Additionally, the final wash procedure involved washing the slides with mRNA Wash Buffer 1 followed by two washes with mRNA Wash Buffer 2 (15 inversions for each wash with replacement of liquid with fresh Wash Buffer 2 in between washes).

### 2.10. Reactivity and Specificity for Unencapsulated mRNA

Specificity was analyzed in terms of S/B at a sample concentration of 20 µg/mL of monovalent naked mRNA. A capture oligo was considered specific if the S/B ratio generated for the target mRNA construct was ≥3, and the S/B ratio generated for all off-target mRNA constructs was <3. 

### 2.11. Precision and Accuracy Testing for Unencapsulated mRNA

Precision and accuracy testing was performed on three slides using an 8-point calibration curve of quadrivalent naked mRNA comprising 7 standards prepared from 0.8 to 15 µg/mL in each construct plus a buffer-only blank. The calibration curve was fit to a quadratic regression for all capture oligos (R^2^ ≥ 0.99), except for D(i), which was fit to a 4PL regression (R^2^ ≥ 0.99). Eight quadrivalent replicates per slide at 6 µg/mL were quantified against the calibration curve and evaluated in terms of %RSD (precision) and % expected (accuracy) in the same manner as the encapsulated mRNA analysis.

## 3. Results

### 3.1. VaxArray mRNA Assay Overview

**Figure 1:**
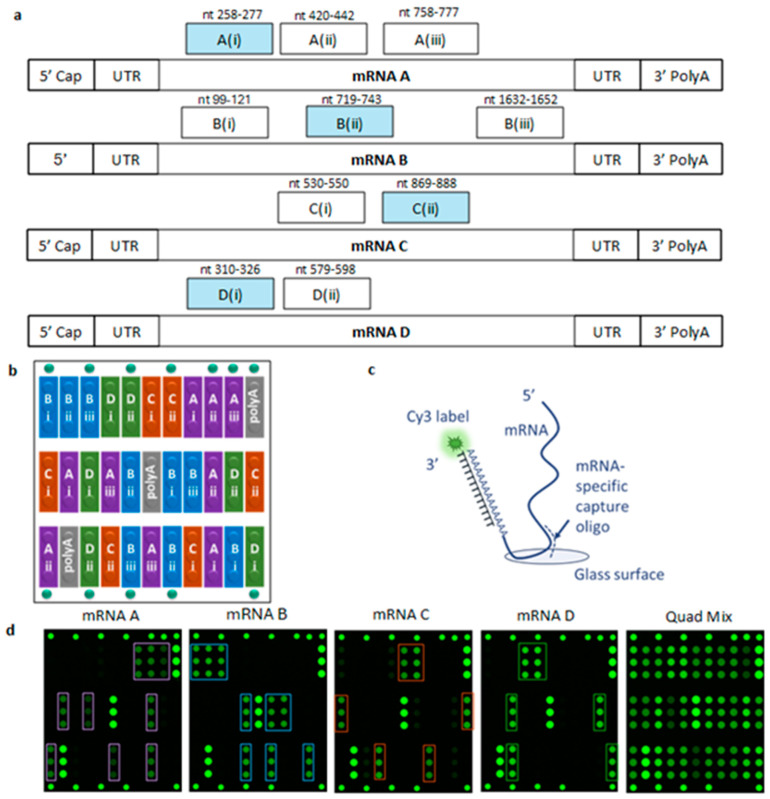
(**a**) Schematic of each mRNA construct with naming and binding locations of the capture oligos. Performance results for blue-shaded capture oligos are the focus of this work. (**b**) Microarray layout where each capture oligo was printed in triplicate in three locations on the microarray (n = 9 spots). (**c**) Schematic of the assay detection principle, in which each printed capture oligo can bind an mRNA construct in a sequence-specific manner and be fluorescently labeled via the polyA tail. (**d**) Representative fluorescence images of each monovalent encapsulated mRNA construct captured and labeled on the microarray, as well as a quadrivalent mixture of the four mRNA constructs, each at a sample concentration of 20 µg/mL. The relevant capture oligos are highlighted for each monovalent sample, with brighter green indicating a higher fluorescence signal.

As described in the Methods section, multiple capture oligos were designed to be specific to four monovalent mRNAs, mRNA A, B, C, and D, encoding four different proteins. All of the candidate capture oligos were screened for reactivity with and specificity for the four mRNAs of interest, and regardless of sequence similarity, capture oligos that were reactive with multiple mRNA constructs, whether due to secondary/tertiary structures or other factors, were not carried forward. The encapsulated mRNA constructs (encapsulated mRNA) were evaluated as a proxy for a multivalent drug product formulation, and the naked mRNAs assessed represented the performance of the drug substance by the VaxArray assay. The capture oligos were printed in microarray format on a glass microscope slide, and the sequence regions targeted by each capture oligo and associated naming are shown in Figure 1a. The results reported here are specifically those from the capture oligos highlighted in blue for each mRNA construct in Figure 1a, namely, A(i), B(ii), C(ii), and D(i).

For each capture oligo within a single microarray, three groups of triplicate spots (n = 9 of each oligo) were printed at different locations throughout the microarray in the orientation detailed in Figure 1b to account for any potential slide surface defects or location-based anomalies. 

The VaxArray mRNA assay detection principle is shown in Figure 1c. Briefly, samples were incubated on the microarray to enable sequence-specific binding to their respective capture oligos. After washing, the fluorescently conjugated detection label targeting the polyA tail was incubated on the microarray to label the polyA tail of all the captured mRNA constructs present. All slides were fluorescently imaged using the VaxArray Imaging System, and the signal intensity of each spot was evaluated. The assay time to result was <2 h and required no additional processing steps such as nucleic acid extraction for encapsulated mRNA.

Representative fluorescence images from the four monovalent encapsulated mRNA constructs and a quadrivalent mixture of the encapsulated mRNA constructs at 20 µg/mL are shown in Figure 1d. Each monovalent image qualitatively depicts the specificity of the assay, as a signal significantly above the background only appears on the capture oligos that target the given mRNA construct. The signal intensities within the images also show that different capture oligos produced different signal intensities, likely due to different sequence-specific binding affinities. Additionally, the relative signal intensities between the monovalent and quadrivalent images are similar. 

To ensure mRNA binding during assay processing steps, the gray-shaded locations labeled ‘polyA’ in Figure 1b contain a 30-mer polyA capture oligo as a universal positive control for all mRNAs containing a 3′ polyA tail. As shown in Figure 1d, signal intensity is observed on these positive control spots for all four constructs, indicating appropriate solution conditions for binding mRNA. While this capture and label are both targeting the polyA tail, the length of the polyA tail enables the simultaneous binding of both the polyA capture and the detection label to act as a positive control.

### 3.2. Assay Is Highly Reactive and Specific for mRNA Encapsulated in LNPs and Exhibits Similar Mono- and Quadrivalent Signal Responses 

Specificity testing was performed by analyzing the four monovalent encapsulated mRNA constructs at 5 µg/mL to gauge whether the capture oligos were binding to their intended mRNA constructs and did not exhibit cross-reactivity with the other constructs. As shown in Table 1, each capture oligo exhibited strong reactivity (S/B ≥ 11) with its target mRNA construct and no reactivity above background (S/B = 1) for all off-target mRNA constructs. The high specificity indicates the suitability of the assay for identity testing and that each mRNA in a multivalent mixture can be assessed independently.

**Figure 2:**
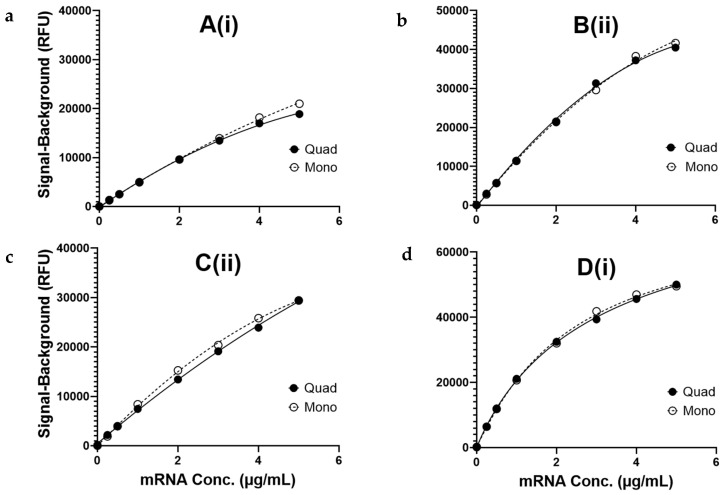
(**a**–**d**) Response curves for monovalent (mono) and quadrivalent (quad) mRNA constructs encapsulated in LNPs on the capture oligos shown: (**a**–**c**) were fit to a quadratic regression (R^2^ ≥ 0.99) for quantification, and (**d**) was fit to a 4-parameter logistic regression (4PL) (R^2^ ≥ 0.99).

A seven-point dilution series from 5.0 to 0.25 µg/mL plus a buffer-only blank was prepared for each monovalent encapsulated mRNA construct and compared to a mixture of the four constructs at concentrations equal to those in the corresponding monovalent samples. The results in Figure 2 demonstrate that the serial dilution of the mRNAs exhibited concentration-dependent signal responses and that response curves for the monovalent materials are like the quadrivalent mRNA mixture, indicating that the assay is free of interference between multiple mRNAs. These data, combined with the specificity shown above, indicate that the VaxArray mRNA assay is suitable for the quantification of both monovalent and multivalent encapsulated mRNAs.

### 3.3. Assay Exhibits Analytical Sensitivity < 1 µg/mL and a ≥100-Fold Quantification Range for mRNA Encapsulated in LNPs

The ULOQ and LLOQ were initially estimated by testing a 16-point dilution series of each monovalent encapsulated mRNA construct and determining the upper and lower ends of the signal response range. These metrics were verified by quantifying four replicates of various dilutions of multivalent encapsulated mRNA near the approximate ULOQ and LLOQ. The LLOQ replicates were tested against a quadrivalent calibration curve that ranged from 0.025 to 1.5 µg/mL, whereas the calibration curve used to quantify the ULOQ samples ranged from 1.25 to 25 µg/mL. The ULOQ and LLOQ were defined as the highest and lowest concentration, respectively, at which the precision of the replicates was <20% RSD, and accuracy was within 100% ± 20% expected. The ULOQ, LLOQ, and quantification range determined for each relevant capture oligo are shown in Table 2, highlighting the range of concentrations, from the LLOQ to the ULOQ (µg/mL), over which the assay can be used quantitatively.

As shown in Table 2, the LLOQ is at least 0.1 µg/mL or lower for all four encapsulated mRNA vaccine components for the capture oligos highlighted. Note that a typical mRNA concentration in a multivalent mRNA vaccine drug product is 100 µg/mL [15], and therefore these quantitation limits are vaccine-relevant. The ULOQs are in the mid-µg/mL range for all four targets, with an overall quantification range of at least 100-fold. Given that each capture oligo exhibited similar reactivity for mono- and quadrivalent formulations, as shown in Figure 2, these limits of quantification are expected to remain consistent for any combination of monovalent encapsulated mRNA constructs tested. 

### 3.4. Assay Exhibits Precision of ≤10% RSD and Accuracy of 100% ± 10% Expected for Encapsulated mRNA 

The quantification of replicate encapsulated mRNA samples against an encapsulated mRNA calibration was performed to gauge the precision and accuracy of the assay. An eight-point calibration curve of quadrivalent encapsulated mRNA (seven standards and a buffer-only blank) from 0.25 to 5 µg/mL of each mRNA construct (all four constructs at equal concentration) was analyzed on each of three slides. Eight replicates of a quadrivalent encapsulated mRNA check standard (2 µg/mL of each mRNA) were quantified on each slide to determine the within-experiment precision and accuracy (Table 3) of the assay. 

Each capture oligo resulted in a precision of ≤10% RSD and accuracy of 100% ± 10% expected over the 24 replicates, indicating that an encapsulated mRNA sample of an unknown concentration could be reproducibly quantified. This performance is within the expected precision and accuracy for the absolute quantification of nucleic acids using qRT-PCR [26]. While UV/Vis measurements are generally capable of precision <5% RSD [27], they lack specificity and cannot quantify individual mRNAs in a mixture of constructs.

### 3.5. Assay Can Be Used Upstream to Identify and Quantify Naked mRNA 

While our focus herein was on demonstrating performance for a real-world example of a quadrivalent, encapsulated mRNA vaccine formulation, the ability of the assay to quantify unencapsulated (naked) mRNA was also assessed. 

Monovalent naked mRNA constructs were tested in a single replicate at a concentration of 20 µg/mL, and the S/B ratio was analyzed to determine the specificity of each capture oligo (Table 4). Consistent with the encapsulated mRNA, each capture oligo was highly reactive and specific to its intended target mRNA construct, with off-target constructs having an S/B ratio of 1. While only for a single replicate herein, these data suggest the applicability of the assay for identity testing of bioprocess-relevant materials.

**Figure 3:**
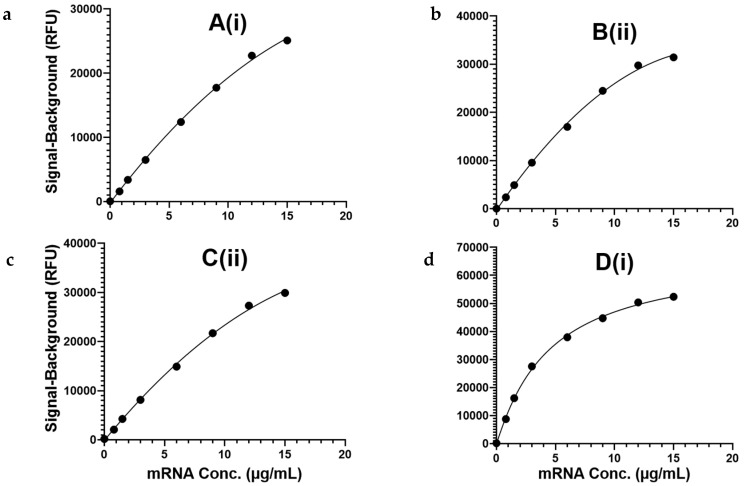
(**a**–**d**) Representative calibration curves from a quadrivalent mixture of naked mRNA are shown for each capture oligo: (**a**–**c**) were fit to a quadratic regression with R^2^ ≥ 0.99, and (**d**) D(i) was fit to a 4PL regression with R^2^ ≥ 0.99.

To approximate the working range as well as assess the precision and accuracy of the naked mRNA constructs, a similar analysis to that undertaken for the encapsulated mRNA was performed. Briefly, each of the three slides was processed with each slide containing an eight-point calibration curve of quadrivalent naked mRNA (seven standards and a buffer-only blank) from 0.8 to 15 µg/mL and eight replicates of a quadrivalent naked mRNA check standard at 6 µg/mL. Representative calibration curves for each capture oligo are shown in Figure 3. Due to the difference in wash procedure and the lack of detergent in the dilution buffer when testing the naked mRNA, there was a difference in overall signal response between the encapsulated and naked mRNA; however, the assay was specific and quantifiable within a vaccine-relevant range.

Accuracy (% expected) and precision (%RSD) results are shown in Table 5. Each capture oligo resulted in a precision of ≤16% RSD, with an accuracy of 100% ± 19% expected. 

## 4. Discussion

The existing analytical tools for mRNA vaccine development were adopted rapidly out of the crucial need for vaccines to reduce morbidity and mortality during the COVID-19 global pandemic, with Herculean efforts undertaken to both develop and characterize these critical vaccines in record time. Many new analytical methods for mRNA characterization are still nascent, as the entire mRNA vaccine landscape along with requirements and regulatory expectations for related CQAs are still in flux. Moreover, an increasing number of novel multivalent mRNA vaccines currently in development or clinical trials pose a unique challenge to existing analytical methods. 

The results reported herein highlight that the VaxArray mRNA assay described can be used for the identity testing and quantification of both naked and mRNA encapsulated in LNPs in a real in-development vaccine drug product. Importantly, the assay is multiplexed with the ability to specifically identify and quantify multiple components in the same sample, maximizing applicability to increasingly prevalent multivalent mRNA vaccines. We acknowledge that quantification via this method is not necessarily a measure of full-length mRNA content given the detection scheme shown in Figure 1c. Depending on the location of the capture sequence along the construct, mRNAs that may be fragmented in a region of the mRNA that is not between the capture and labeling regions would still be captured with this method. This presents an interesting design choice in that capture sequences designed closer to the 5′ end can give a better indication of how much mRNA is unfragmented, and potentially comparing quantification of mRNA based on different capture oligos may be able to provide information regarding mRNA fragmentation.

As mentioned, the current methods for identity confirmation and quantification in multivalent samples are typically sequencing-based [19]. While sequencing-based methods inherently provide high information content [23,28], the up-front sample extraction and library preparation often required, as well as the downstream bioinformatics and data analysis needed, may make sequencing methods overly complicated for routine identity and quantity measurements. In contrast to sequencing-based methods that provide full sequence identity, VaxArray mRNA identity and quantity assays do not require any up-front sample processing other than a rapid lysis step and provide the needed specificity in multivalent mixtures with a similar information content to RT-PCR-based methods. And while RT-PCR is fast, affordable, and sensitive, it typically lacks the ability to be highly multiplexed with ease. Higher information content that would be difficult to achieve with an RT-PCR-based approach can be achieved with a VaxArray assay by adding more capture sequences per construct. In addition, VaxArray does not require the engineering controls typically utilized in RT-PCR-based approaches because there is no amplification step, and it can instead be run on a standard benchtop with minimal workflow and engineering controls.

Importantly, as new influenza strains are recommended for vaccine inclusion due to antigenic drift, the VaxArray mRNA assay can be updated within several weeks and can therefore be impactfully used during a seasonal vaccine manufacturing campaign. The steps to design and add new capture oligos to the microarray and test the new sequences for reactivity and specificity to the new mRNA target(s) can be performed in parallel with mRNA vaccine optimization and formulation. Alternatively, capture sequences can be designed to target regions of the mRNA corresponding to conserved portions of the antigen of interest to enable a variety of closely related mRNAs to be captured as a function of antigenic drift to enable the use of the same assay over time. 

Because VaxArray is a platform solution, assays can be developed for multivalent vaccines within a few months, such as those targeting protection against multiple pathogens like quadrivalent influenza plus COVID-19 [14]; respiratory syncytial virus (RSV) and human metapneumovirus [29]; or influenza, RSV, and COVID-19 [30]. In addition, the analysis of higher-valency mRNA mixtures in development [13] is easily achievable as vaccines increase in complexity to include additional antigens. 

## 5. Conclusions

As the mRNA vaccine and therapeutic industries are rapidly advancing and evolving, it is vital that next-generation analytical technologies allow for the identification and quantification of each unique mRNA within a multivalent mixture throughout the vaccine manufacturing process. The VaxArray mRNA assay can be rapidly developed and customized for each individual mRNA in a vaccine, for accurate and precise quantification of both naked and encapsulated mRNA formulations. Given the benefits discussed, we hope the VaxArray technology can help fill a gap in current mRNA analytics for multivalent vaccines.

## Figures and Tables

**Table 1:** Reactivity and specificity of monovalent mRNA constructs encapsulated in LNPs at 5 µg/mL.

Capture Oligo	Signal to Background Ratio (S/B) ± σ (n = 8 Replicates)			
	mRNA A	mRNA B	mRNA C	mRNA D
A(i)	11 ± 1	1 ± 0	1 ± 0	1 ± 0
B(ii)	1 ± 0	13 ± 1	1 ± 0	1 ± 0
C(ii)	1 ± 0	1 ± 0	14 ± 0	1 ± 0
D(i)	1 ± 0	1 ± 0	1 ± 0	22 ± 1

**Table 2:** Analytical sensitivity and range for mRNA encapsulated in LNPs.

Capture Oligo	Lower Limit of Quantification (LLOQ) (µg/mL)	Upper Limit of Quantification (ULOQ) (µg/mL)	Quantification Range (ULOQ/LLOQ)
A(i)	0.1	≥20	≥200
B(ii)	≤0.05	≥20	≥400
C(ii)	≤0.05	≥20	≥400
D(i)	≤0.05	5	≥100

**Table 3:** Precision and accuracy of mRNA encapsulated in LNPs.

Capture Oligo	Precision (%RSD)				Accuracy (% Expected)			
	Slide 1 (n = 8)	Slide 2 (n = 8)	Slide 3 (n = 8)	All Slides (n = 24)	Slide 1 (n = 8)	Slide 2 (n = 8)	Slide 3 (n = 8)	All Slides (n = 24)
A(i)	5%	6%	6%	8%	91%	93%	103%	96%
B(ii)	4%	3%	3%	5%	94%	100%	102%	99%
C(ii)	4%	5%	4%	6%	96%	93%	102%	97%
D(i)	9%	6%	6%	10%	90%	100%	106%	99%

**Table 4:** Reactivity and specificity of naked mRNA at 20 µg/mL.

Capture Oligo	Signal to Background Ratio (S/B)			
	mRNA A	mRNA B	mRNA C	mRNA D
A(i)	14	1	1	1
B(ii)	1	13	1	1
C(ii)	1	1	10	1
D(i)	1	1	1	16

**Table 5:** Precision and accuracy (naked and unencapsulated mRNA).

Capture Oligo	Precision (%RSD)				Accuracy (% Expected)			
	Slide 1 (n = 8)	Slide 2 (n = 8)	Slide 3 (n = 8)	All Slides (n = 24)	Slide 1 (n = 8)	Slide 2 (n = 8)	Slide 3 (n = 8)	All Slides (n = 24)
A(i)	4%	6%	10%	8%	97%	95%	105%	99%
B(ii)	7%	7%	8%	7%	99%	102%	98%	100%
C(ii)	5%	7%	8%	8%	98%	97%	107%	101%
D(i)	9%	14%	14%	16%	93%	81%	105%	93%

## Data Availability

All relevant data from this study are available from the corresponding author.
